# Cerebrospinal Fluid Homer-3 Autoantibodies in a Patient with Amnestic Mild Cognitive Impairment

**DOI:** 10.3390/brainsci13010125

**Published:** 2023-01-11

**Authors:** Niels Hansen, Katrin Radenbach, Kristin Rentzsch, Janosch Fox, Jens Wiltfang, Claudia Bartels

**Affiliations:** 1 Department of Psychiatry and Psychotherapy, University Medical Center Goettingen, Von-Siebold-Str. 5, 37075 Goettingen, Germany; 2 Translational Psychoneuroscience, University Medical Center Goettingen, Von-Siebold-Str. 5, 37075 Goettingen, Germany; 3 Clinical Immunological Laboratory Prof. Stöcker, 23627 Groß Grönau, Germany; 4 German Center for Neurodegenerative Diseases (DZNE), Von-Siebold-Str. 3a, 37075 Goettingen, Germany; 5 Neurosciences and Signaling Group, Institute of Biomedicine (iBiMED), Department of Medical Sciences, University of Aveiro, 3810-193 Aveiro, Portugal

**Keywords:** Homer-3 autoantibodies, cerebellum, cognition, mild cognitive impairment

## Abstract

(1) Background: Homer-3 antibodies are associated with cerebellar disease ranging from subacute degeneration to cerebellitis. However, cognitive impairment associated with Homer-3 autoantibodies has not been reported until now. (2) Methods: in retrospect, we systematically studied clinical, cranial magnetic resonance imaging (cMRI), electroencephalography (EEG) and lumbar puncture data, including neural autoantibodies of a clinical case. (3) Results: we describe the case of a 56-year-old woman presenting with amnestic mild cognitive impairment in association with serum and CSF detection of Homer-3 autoantibodies and a depressive syndrome. cMRI revealed cerebellar atrophy. CSF analysis showed elevated ptau181 protein. Applying the criteria for an autoimmune psychiatric syndrome revealed a plausible autoimmune basis for the mild cognitive impairment. (4) Discussions: our case report demonstrates an amnestic mild cognitive impairment and depressive symptoms associated with Homer-3 autoantibodies as a novel feature of Homer-3 antibody-related disease. We also propose that cognitive dysfunction might result from impaired AMPAR signaling in the hippocampus induced by Homer-3 antibodies, which will have to be verified in further research.

## 1. Introduction

Homer-3 is a protein at the post-synapse of Purkinje cells in the cerebellum that connects the metabotropic glutamate receptor 1 (GluR1) to the calcium channel inositol 1,4,5-trisphosphate receptor type 1 [1]. The main phenotype observed in six patients with Homer-3 antibodies is the subacute cerebellar ataxia. Less common phenotypes with encephalopathy, rapid-eye-movement (REM) sleep behavioral disorder, autonomic disturbances or myeloradiculopathy have also been reported ([2], Table 1). Another recent study showed that Homer-3 can regulate the response element in T-cells through their enabled vasodilatator-stimulated phosphoprotein (Ena/VASP) homology 1 (EVH1) domain [3] suggesting a potential link between Homer-3 autoantibody-mediated effects on T-cell immunopathology. Here we report the case of a woman with predominantly amnestic mild cognitive impairment (MCI) in addition to a depressive syndrome so far not reported, as she presented Homer-3 autoantibodies associated with autoimmune encephalitis.

## 2. Case Report

A 56-year-old woman presented in our hospital because of sleep disturbances, memory complaints, concentration deficits, social withdrawal and mood disturbances often accompanied by anger and petulance. Her medical history reveals an epileptic seizure in 2000 under prothipendyl medication, and she had bilateral carpal tunnel syndrome. She also suffered from hypothyreosis, sleep apnea syndrome and lumbar disc protrusions. She was admitted to our geriatric psychiatric unit due to progressive memory decline. A first depressive episode had emerged after her divorce 27 years ago, followed by two more episodes 17 and 4 years ago. Prior antidepressant medication comprised various antidepressants such as paroxetine, mirtazapine, moclobemide, trimipramine, doxepin and agomelatine. She had had many outpatient and inpatient contacts with consultant psychiatrists. This patient has six siblings, two of whom also suffer from a major depressive disorder. Her family history was also positive for Alzheimer’s dementia (father). She was married and divorced twice and delivered two children during her first marriage. She has a basic education level (primary and secondary/high school) and worked as a professional hairdresser. At inpatient admission, psychopathological examination revealed a depressive syndrome with depressive mood, anhedonia, loss of drive and social withdrawal (diagnosed as a recurrent major depressive disorder, moderate; ICD-10: F33.1). At the time-point of neuropsychological testing, depressive symptoms had already subsided with a Montgomery–Åsberg Depression Rating Scale score indicative of depressive symptoms of only mild severity (MADRS score: 8/60; Table 2). 

**Figure 1:**
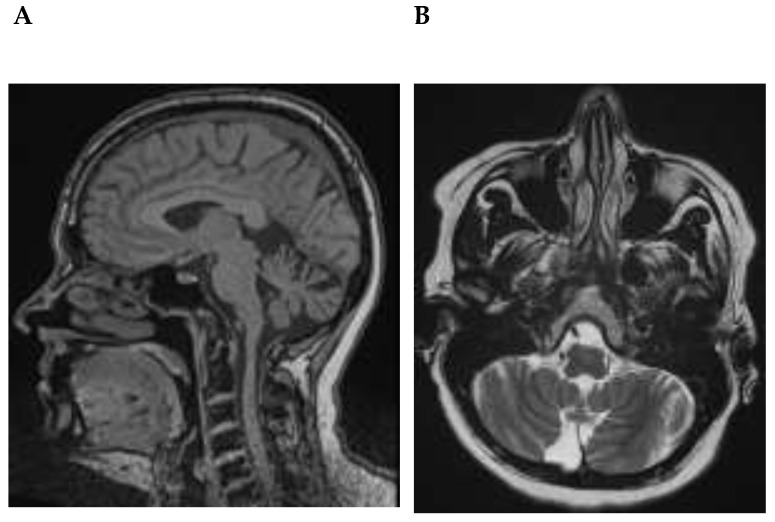
Cerebellar atrophy in a patient associated with Homer-3 autoantibodies. (**A**) Sagittal T1-weighted magnetic resonance imaging (MRI) and (**B**) transversal T2-weighted MRI showing cerebellar atrophy.

Cognitive screening revealed mild cognitive deficits (MMST score: 24/30). Neuropsychological testing showed impairments most pronounced in executive functions (semantic and phonematic word-fluency cognitive flexibility, action planning) and memory (working memory, figural and verbal memory; supplementary Figure S1), altogether concurring with a syndromal diagnosis of an amnestic MCI in multiple domains. Her physical examination revealed no relevant pathology of the organs, skin or musculoskeletal system. Moreover, her neurological examination was unremarkable apart from the aforementioned cognitive deficits. Brain magnetic resonance imaging (MRI) showed signs of cerebellar atrophy (Table 2, Figure 1A,B). 

Lumbar puncture revealed elevated phosphorylated tau protein 181 (ptau 181; Table 2). Elevated tau protein was confirmed in an external reference laboratory (Table 2). We sought autoantibodies against paraneoplastic antigens such as anti-glutamic acid decarboxylase 65 (GAD65), Zic4, -TR/DNER, SOX1, Ma2, amphiphysin, CV2, Ri, Yo and HuD and against membrane-surface antigens such as the anti-N-methyl D-aspartate receptor (NMDAR), -Leucin rich glioma inactivated protein 1 (LGI1), -α-amino-3-hydroxy-5-methyl-4-isoxazolepropionic acid receptor 1/2 (AMPAR1/2), -gamma-aminobutyric acid B receptor1-/2-(GABARB1/2), -dipeptidyl-peptidase-like protein-6 (DPPX), and -contactin-associated protein-2 (CASPR2) autoantibodies to rule out different types of autoantibody-mediated autoimmune encephalitis in our biomaterial probes. Biomaterial probe analyses were positive for anti-Homer-3 antibodies in serum (1:320) and cerebrospinal fluid (CSF) (1:3.2; Table 2) anti-neural antigen IgG immunofluorescence testing with BIOCHIP-mosaic with brain tissue and recombinant cells. We diagnosed autoimmune-based MCI together with depressive symptoms according to the Hansen et al. clinical criteria [10] (Table 3), because she fulfilled the criteria for a probable autoimmune-based psychiatric syndrome, and IgG Homer-3 autoantibodies were detected in her CSF. She thus presented a definitive autoimmune-based psychiatric syndrome diagnosis according to the Hansen et al. criteria [10]. However, criteria for definitive autoimmune limbic encephalitis were not met [11] (Table 3). 

We had considered a neurodegenerative disorder as a differential diagnosis since we had identified elevated ptau181 and because of her cognitive impairment. However, the time course entailing rapidly developing severe cognitive dysfunction along with depression may correspond to a rapidly progressing neurodegenerative disorder such as Alzheimer’s disease or another rapidly worsening neurodegenerative disorder. Nevertheless, other clinical and laboratory evidence is lacking to suggest such a disease. As we failed to detect a reduced CSF Aß42/40 ratio or diminished Aß42 in the CSF, an Alzheimer’s diagnosis was not supported by CSF biomarkers according to recent guidelines [12]. We suggested additional diagnostics such as a tumor search via a whole-body positron emission tomography (PET) scan and individual therapy trial. However, the patient declined further diagnostics and immunotherapy. We prescribed bupropion as antidepressant medication and omitted sertraline. She also underwent repetitive transcranial magnetic-stimulation treatment, but this failed to reveal any objective improvement.

## 3. Discussion

With this case report, we aim to expand the clinical spectrum of adult anti-Homer-3 autoantibody-associated disease by reporting MCI in association with Homer-3 antibodies. Anti-Homer-3-associated disease involving subacute cognitive impairment lasting two weeks has been reported in children [7]. Our patient met the criteria for definitive autoimmune cognitive dysfunction according to the Hansen et al. criteria [10]. Homer-3 autoantibody-associated disease has mainly been identified in conjunction with a phenotype mimicking cerebellar multisystem atrophy [13], cerebellar volume anomalies in cerebellitis [14,15], subacute idiopathic ataxia [9], subacute cerebellar degeneration [8], and “Medusa head ataxia” [6] (Table 1). Thus, the primary location of the proposed immunopathology of Homer-3 antibodies is the cerebellum, and to our knowledge, there are no other published findings of the occurrence/detection/presence of Homer-3 antibodies in adult cognitive impairment. It is tempting to postulate a link between the cerebellum, Homer-3 autoantibodies, and cognitive impairment in our patient, although we cannot unequivocally prove it. Our patient’s cerebellar atrophy is further evidence of her cerebellum’s structural pathology. There is published evidence that the cerebellum contributes to cognitive function in various disease states, through impaired cerebro-cerebellar circuit integrity [2], through the association between cerebellar cortex thickness on MRI and cognitive impairment, and in autoimmune CNS diseases such as multiple sclerosis [16]. In addition to the cerebro-cerebellar circuitry, the interaction between the cerebellum and hippocampus may be playing an important role in our patient’s cognitive impairment. The cerebellum and hippocampus interact in enabling cognitive processes [17]. There are therefore two possibilities [via a (1) cortical or (2) hippocampal connection] for how a cerebellar immune process induced or triggered by Homer-3 autoantibodies could affect cognition. Interaction between the cerebellum and cognitive function may eventually reveal that Homer-3 antibody disease can also trigger cognitive impairment, with primary neuroinflammation in cerebellar structures likely. Homer-3 is a protein involved in regulating AMPAR signaling in the hippocampus in mice and is associated with learning and memory [18]. Considering their animal study, it is tempting to postulate a role for Homer-3 antibodies in the hippocampus also, and not just in the cerebellum in a memory-and cognitive-dysfunction context. However, the pathophysiology of exactly how Homer-3 antibodies are associated with an autoimmune process in the cerebellum is completely unclear. Because Homer-3 antibodies are involved in elements of the T-cell response, their blockade should alter the T-cell responses that might trigger autoinflammation. However, this hypothesis is speculative and requires more investigation.

### Limitations

This case report is limited by our speculation about the location in the brain where autoantibodies occur, as no brain tissue specimen was removed and later analyzed. Moreover, the case presented here also offers alternative or multifactorial explanations for the observed cognitive impairment, such as the impact of depression on cognition, even at a subsyndromal level. Therefore, the interplay between the presence of Homer-3 antibodies, depressive symptomatology, and cognitive impairment remains exploratory at the level of clinical clues and does no rely on the partially fulfilled, revised Witebsky criteria [19] for causing an autoimmune disease. Our report provides no evidence of pathogenicity resulting from the transfer of these autoantibodies or T cells from our patient to animals. Furthermore, we have not replicated cognitive dysfunction in animal experiments, which could deliver additional evidence for the autoantibodies’ causality. The pathogenicity of autoantibodies in psychiatric disorders is unclear, and there is no clear causal relationship when applying the Witebsky criteria in psychiatric patients who present with neural autoantibodies. Our patient partially fulfilled the Witebsky criteria [20], i.e., a definitively identified antigen and circulating autoantibodies active at body temperature. Couthino discussed the criteria for such an antibody-mediated psychiatric disorder in a review paper [21] and puts into perspective the preliminary though plausible evidence of autoimmunity in autoimmune encephalitis based on Graus et al. criteria [11]. As our CAP criteria are even less able of fulfilling such a Witebsky postulate, they should be interpreted with caution. Further animal–human transfer studies are needed to gain more insight into the pathogenicity of Homer-3 autoantibodies in cognitive dysfunction and to understand the relationship between autoimmunity and neuropsychiatric disorders. 

## 4. Conclusions

Taken together, our report extends the clinical spectrum of anti-Homer-3-associated disease to include adult cognitive impairment. However, we cannot rule out that MCI risk factors such as depressive disorder or sleep apnea syndrome may have contributed to the patient’s cognitive impairment. No other risk factors (i.e., alcohol abuse) were identified. Further studies in large patient cohorts presenting these antibodies should be conducted to confirm our findings and reveal more insights in order to enable improved diagnostics and therapeutic approaches. In light of our patient’s relatively young age and her neuropsychiatric symptoms’ time course and severity, a neurodegenerative disorder as the cause of her symptoms is unlikely. The patient’s prognosis will likely be improved by the probable long-term clinical benefit of immunotherapy, as a recent series by Liu et al. noted [2], provided the patient chooses immunotherapy over time.

## Figures and Tables

**Table 1:** Homer-3 autoantibodies in neuropsychiatric disease: case report and series.

Neuropsychiatric Disease	References
Autonomic disturbances	[2]
Cerebellitis	[4]
Dysfunctional emotional control	[5]
Encephalopathy	[2]
Medusa head ataxia	[6]
Myeloradiculopathy	[2]
Pancerebellar syndrome and encephalopathy	[7]
REM sleep behavioral disorder	[2]
Subacute cerebellar ataxia	[2,5]
Subacute cerebellar degeneration	[8]
Subacute idiopathic ataxia	[9]

Abbreviations: REM = rapid eye movement.

**Table 2:** Clinical and laboratory characteristics of patient.

Parameter	Data
**Demographic parameter**	
Sex	f
Age y	56
Age of onset y	54
**Psychopathology**	
Orientation dysfunction	1
Attentional dysfunction	1
Memory disturbances	1
Formal thought disorder	1
Affective disturbance	1
Drive and psychomotor disturbance	1
**CSF**	
Cell count (<5 µL)	0
Albumin mg/L	261
NSE (<30 ng/mL)	24.1
S100 (<2.7 µg/L)	1.6
Tau protein (<450 pg/mL)	128
P tau protein 181 (<61 pg/mL)	70
Aß42 (>450 pg/mL)	1539
Aß40	19,275
Ratio Aß42/40 × 10 (>0.5)	0.8
Blood–brain barrier disturbance	Not present
Intrathecal IgG synthesis	Not present
Serum	
NSE (<30 ng/mL)	22.8
S100 (<1.5 µg/L)	0.06
**MRI**	
Generalized atrophy	0 *
Focal atrophy	1 #
Hippocampal atrophy	0
Vascular pathology	0

Abbreviations: Aß42 = amyloid-ß 42, Aß40 = amyloid-ß 40, CSF = cerebrospinal fluid, MRI = magnetic resonance imaging, p-tau protein 181 = phosphorylated tau protein 181, ratio Aß42/40 = ratio of amyloid-ß 42/ratio of amyloid-ß 40, y = years. The values are depicted as mean ± standard deviation. For laboratory data normal ranges are shown in brackets. Reference values: refers to reference values from the Neurochemistry Laboratory, Neurology Department, University Medical Center Göttingen. * 0 = item not present, # 1 = item present.

**Table 3:** Criteria for the autoimmune genesis of the symptomatology.

I Definitive limbic autoimmune encephalitis according to Graus et al. [11]	
**Criterion**	**Completion of Criterion**
A. Subacute presentation of cognitive dysfunction with working memory deficits together with psychiatric symptoms (depressive symptoms)	Yes
B. T2/FLAIR MRI signal changes in temporal lobe	No
C. CSF pleocytosis or temporal EEG abnormalities	No
D. Detection of neural autoantibodies in serum and CSF	Yes
E. Exclusion of other reasons	Yes
Sum	3 of 4 required criteria = no definitive limbic autoimmune encephalitis
II Definitive autoimmune based psychiatric syndrome according to Hansen et al. [10]	
**Criterion**	**Completion of Criterion**
A. Minor cognitive impairment	Yes
B. Severe cognitive dysfunction	Yes
C. Two items: elevated ptau181 and presence of serum Homer-3 autoantibodies	Yes
D. Presence of CSF IgG Homer-3 autoantibodies	Yes
Sum	4 of 4 required criteria = definitive autoimmune based psychiatric syndrome

## Data Availability

The data are available on request from the corresponding author.

## Supplementary Materials

The following supporting information can be downloaded at: https://www.mdpi.com/article/10.3390/brainsci13010125/s1. Figure S1: Neuropsychological profile. Illustration of cognitive test results presented as z-scores. The area between dotted lines denotes the normal range.

## Abbreviations

CERAD: Consortium to Establish a Registry for Alzheimer’s Disease; WMS IV: Wechsler Memory Scale—4th edition; RCFT: Rey-Osterrieth Complex Figure Test; WAIS-IV: Wechsler Adult Intelligence Scale—4th edition; TMT: Trail Making Test; MEM: memory; VIS: visuospatial cognition; EXE: executive functions; ATT: attention; LANG: language.
