# Supplementary material for: Cerebrospinal Fluid Homer-3 Autoantibodies in a Patient with Amnestic Mild Cognitive Impairment
Source: Brain Sci. 2023 Jan 11;13(1):125. doi: 10.3390/brainsci13010125 (PMC9856294; doi:10.3390/brainsci13010125)
Supplement: Supplementary file 1 [file brainsci-13-00125-s001.zip › brainsci-2129856-supplementary.pdf]

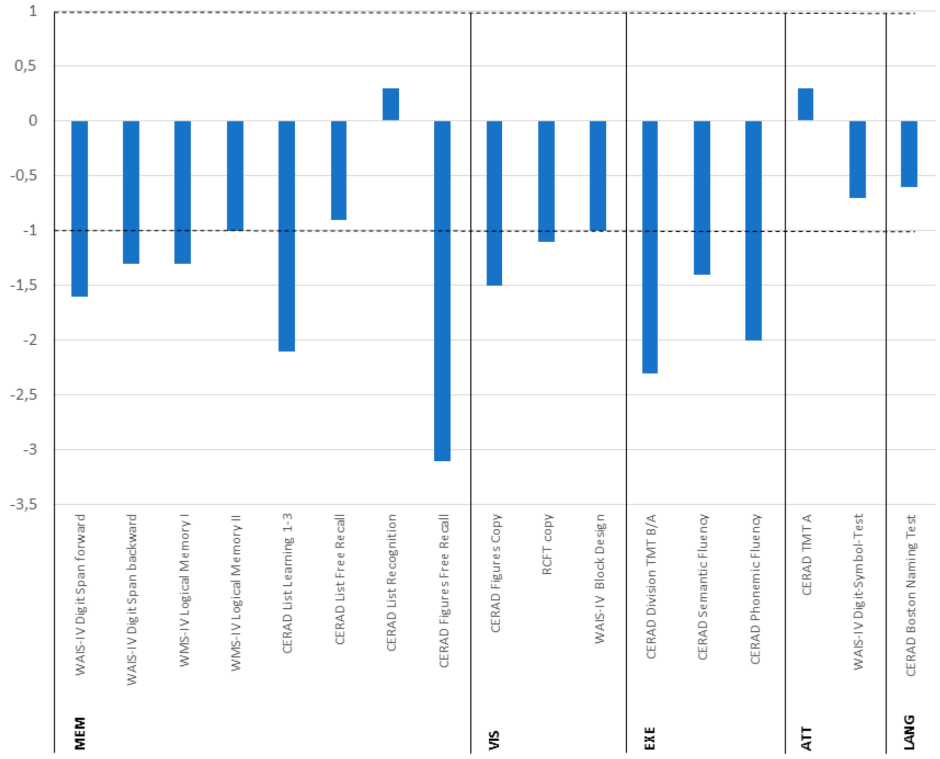

Figure S1. Neuropsychological profile. Illustration of cognitive test results presented as z-scores. The area between dotted lines denotes the normal range.
